# Healing through heritage tourism: indigenous cultural practices and mental wellness in traditional communities

**DOI:** 10.3389/fpsyg.2026.1899219

**Published:** 2026-07-31

**Authors:** Rui Zhao, Jing Zhang, Rui Li

**Affiliations:** School of Economics and Management, Wenshan University, Wenshan, China

**Keywords:** cultural heritage preservation, cultural identity, heritage tourism, indigenous communities, indigenous well-being, psychological resilience, traditional healing practices

## Abstract

**Introduction:**

Heritage tourism emerged as an important mechanism for preserving indigenous cultural traditions while potentially supporting psychosocial well-being. This mixed-methods study examined the associations between heritage tourism participation, cultural identity, resilience, social support, and psychological well-being among indigenous communities in Southwest China.

**Methods:**

A total of 180 participants from Miao, Dong and Naxi communities were recruited using purposive and snowball sampling strategies. Quantitative data were collected using the Connor–Davidson Resilience Scale (CD-RISC-10), WHO-5 Well-Being Index, Multidimensional Scale of Perceived Social Support (MSPSS), Perceived Stress Scale (PSS-10) and Cultural Identity and Belongingness Scale. Qualitative data obtained through semi-structured interviews, focus group discussions and the participant observation.

**Results:**

Reliability analyses demonstrated strong internal consistency across instruments (Cronbach’s *α* = 0.86–0.91). Correlation analyses revealed the significant positive associations between heritage tourism participation and psychological well-being (*r* = 0.38, *p* < 0.01), resilience (*r* = 0.31, *p* < 0.01), social support (*r* = 0.28, *p* < 0.01) and cultural identity (*r* = 0.34, *p* < 0.01). Thematic analysis identified six major themes including cultural participation and resilience, traditional healing and psychological wellness, social cohesion and belongingness, ecological attachment and spiritual restoration, economic empowerment and well-being and tourism-related stress and cultural commodification. Participants described storytelling, ritual practices, traditional healing systems, ecological stewardship and community festivals as important sources of emotional support, cultural continuity and collective resilience.

**Discussion:**

The concerns regarding commercialization, cultural performance pressure and unequal tourism benefits also reported. The findings suggest that heritage tourism may function as culturally grounded resource associated with psychological well-being when implemented through community-led, culturally sensitive and sustainable tourism frameworks.

## 1 Introduction

Recent decades have seen psychological research expand biomedical perspectives of mental health to include social, cultural, environmental and spiritual factors, along with cultural identity, collective memory, traditional knowledge systems and community engagement in promoting emotional well-being (Page-Reeves and McKinney, 2019). Traditions may include spirituality, the environment, social collaboration, rituals and oral traditions and they often serve as psychosocial mechanisms that promote resilience, connection and healing (Zechner et al., 2022). Heritage tourism is a type of travel experience that is found worldwide, in which travel is centered around cultural traditions, historical knowledge, rituals, indigenous lifestyles, artistic expressions, sacred landscapes and community heritage (Santa and Tiatco, 2019).

Indigenous heritage tourism can include tourists participating in traditional ceremonies, storytelling, ecological activities, craft workshops, music performances and spiritual rituals, which can create intercultural dialog and economic development for local communities (Reddy and Sailesh, 2024). The preservation and presentation of indigenous cultural practices in tourism settings may also result in greater community self-esteem, social validation and psychological hardness.

The relationship between cultural identity and mental health has been well documented in the community psychology literature, with strong cultural affiliation associated with lower levels of anxiety, emotional stability, higher self-esteem and lower social alienation (Chang et al., 2025). Traditional customs and collective practices are more likely to be present in communities that exhibit higher levels of social cohesion and intergenerational solidarity and rituals, ceremonies, music, dance and spiritual practices provide symbolic meaning that helps with stress, grief, trauma and social disruption. Heritage tourism could be an area in which indigenous communities reinvigorate traditional knowledge and reinforce group identity (Oflazoğlu and Dora, 2024).

Healing systems of many indigenous cultures are rooted in connections to land, ancestors and ecological relationships and sacred forests, rivers, mountains and ceremonial spaces are a source of emotional equilibrium and spiritual healing (Perriam, 2015). Tourism that protects such environments can lead to experiences that reaffirm cultural continuity and environmental attachment, making cultural landscapes therapeutic spaces in which identity, spirituality and community memory are reasserted, to the mutual benefit of indigenous hosts and visitors who seek culturally significant engagement and emotional replenishment (Lonn, 2025). For indigenous communities, heritage tourism can also cause mental distress, identity conflict and social tensions over heritage (Marbun, 2025).

Unequal economic distribution and external control of tourism resources can also lead to psychological distress and community fragmentation. Although much heritage tourism research has focused on economic sustainability, destination branding, visitor satisfaction and cultural conservation (Perry, 2023), the psychological dimensions of indigenous heritage tourism have not been well-studied, especially the emotional resilience, social belongingness and collective mental wellness of indigenous communities. Existing mental health research in indigenous contexts has often focused on trauma, marginalization and healthcare disparities rather than strengths-based cultural healing mechanisms (Bryant et al., 2021). Indigenous mental wellness in traditional communities and the impact of indigenous cultural tourism practices is a research gap.

This gap is addressed in the present study by looking at the emotional wellbeing that Indigenous peoples experience through participation in heritage tourism via culture, rituals, stories, nature, art forms and community engagement, as well as the psychological implications of tourism-led commodification and cultural transformation (Ruhanen and Whitford, 2021). As globalization has increased the challenges of urbanization, environmental degradation, migration and cultural homogenization, heritage tourism is a socio-economic and psychosocial process in which the empirical evidence of the impacts on Indigenous mental health will allow policymakers, practitioners (psychologists who work with Indigenous populations), developers of tourist destinations and organizations to develop strategies to maintain local control and strengthen resilience (Memon et al., 2021).

## 2 Methodology

### 2.1 Research design and psychological inquiry framework

The current study used a mixed-methods research design that included both qualitative ethnographic methods and quantitative psychosocial assessment tools to examine the intersection of indigenous cultural practices, heritage tourism and mental wellness, as this type of research design can capture both the subjective emotional experiences of participants and measurable psychosocial outcomes related to participation in heritage tourism activities (Li, 2025). The qualitative aspect provided a rich understanding of cultural identity, emotional healing, collective rituals and traditional wellness practices, while the quantitative element could be applied for systematic assessment of resilience, stress reduction, social belongingness and psychological well-being.

The research design used culturally appropriate and community-engaged research methods at every stage of data collection and analysis, included Indigenous communities as collaborative partners rather than research participants, considered emotional well-being in the context of Indigenous worldviews, which is often related to mental wellness and can be linked to spirituality, ecological connectedness, relationships with community and ancestral ties (Christensen, 2012) and was structured to have long-term field engagement (12 months) to gain an understanding of the social and cultural meanings of heritage tourism participation at seasonal ceremonies, tourism events and community interactions (Hollway et al., 2018), with methodological triangulation of interviews, participant observation, focus group discussions and psychosocial questionnaires to strengthen the robustness and interpretive thickness of the results (Gibson, 2017). The design provided a strong foundation for understanding how heritage tourism contributes to emotional resilience, strengthens cultural identity and promotes collective mental wellness in Indigenous communities.

### 2.2 Study setting and indigenous community context

The study was conducted in three indigenous ethnic minority heritage tourism regions in Southwest China including, the Miao communities of Guizhou Province, the Dong communities of southeastern Guizhou and the Naxi communities of Lijiang in Yunnan Province. These communities were selected because they represent culturally significant heritage tourism destinations in China and have successfully preserved rich traditions despite increasing tourism development. The selected regions maintain diverse cultural practices, including ritual ceremonies, storytelling traditions, indigenous ecological knowledge, spiritual healing systems, handicraft production, traditional music, dance performances and community-based tourism activities. The Miao communities are particularly recognized for their vibrant cultural festivals, intricate embroidery traditions, ceremonial music and ritual practices, while the Dong communities are known for their iconic drum towers, folk songs, oral storytelling traditions and handicraft production. The Naxi communities of Yunnan preserve distinctive traditional healing systems, ecological rituals and sacred cultural landscapes that remain central to the community identity and spiritual life.

Prior to data collection, the research team conducted extensive preliminary visits to each study site to establish collaborative relationships with village leaders, cultural custodians, tourism coordinators and traditional healers. Community consultations were undertaken to identify culturally appropriate research procedures, ensure respect for local customs and traditions and obtain community-level consent for participation. Particular attention was given to sacred cultural spaces and ceremonial activities, where access, observation and documentation were strictly guided by community protocols and cultural governance structures. The selected communities represented varying levels of tourism development, ranging from highly established heritage tourism destinations to smaller community-managed tourism initiatives. Specifically, the Miao and Naxi communities exhibited relatively high levels of tourism development, whereas the Dong communities represented a moderate level of tourism engagement. This diversity provided an important opportunity to examine how different levels of heritage tourism participation influenced cultural identity, psychological well-being, social cohesion, emotional resilience and community wellness among indigenous populations while maintaining sensitivity to local cultural values and traditional knowledge systems.

### 2.3 Participant selection and community representation

Purposive sampling was used to recruit participants who were directly involved in cultural preservation and heritage tourism, including traditional healers, storytellers, artisans, ritual practitioners, musicians, dancers, eco-tourism guides, members of women’s cooperatives, youth volunteers and community tourism coordinators, who represented a range of cultural perspectives and psychosocial experiences from indigenous communities (Pai et al., 2026). A total of 180 participants were recruited across the selected study settings; participants had to be at least 18 years of age and had at least 2 years of experience in cultural or tourism activities (Kohsaka and Rogel, 2021). Participants were identified based on their extensive knowledge of culture and experience in traditional practices and participants were also identified through snowball sampling, where community leaders and local coordinators identified participants who were known in their communities for their experience in cultural healing, ecological stewardship and tourism engagement. Table 1 shows demographic characteristics of participants.

**Table 1:** Demographic characteristics of participants (*n* = 180).

Variable	Category	n	%
Gender	Male	84	46.7
	Female	96	53.3
Age	18–30	52	28.9
	31–50	73	40.6
	>50	55	30.5
Community	Miao	60	33.3
	Dong	60	33.3
	Naxi	60	33.3
Tourism participation	Low	40	22.2
	Moderate	72	40.0
	High	68	37.8

To enhance participant diversity and ensure inclusion of culturally knowledgeable individuals who may not have been identified through formal community networks, snowball sampling was further employed whereby initial participants recommended additional community members recognized for their involvement in cultural preservation, traditional healing, ecological stewardship and tourism-related activities (Kohsaka and Rogel, 2021). A total of 180 participants were recruited across the three study locations, including the Miao communities of Guizhou Province, the Dong communities of southeastern Guizhou and the Naxi communities of Yunnan Province. Efforts were made to ensure balanced representation across gender, age groups, levels of tourism participation and cultural roles within each community. Of the total participants, 84 (46.7%) were male and 96 (53.3%) were female. The age distribution included 52 participants (28.9%) aged between 18 and 30 years, 73 participants (40.6%) aged between 31 and 50 years and 55 participants (30.5%) aged over 50 years. Community representation was equally distributed, with 60 participants (33.3%) recruited from each of the Miao, Dong and Naxi communities. Regarding involvement in heritage tourism activities, 40 participants (22.2%) reported low levels of participation, 72 participants (40.0%) reported moderate involvement and 68 participants (37.8%) reported high levels of engagement. This distribution ensured adequate representation of diverse cultural experiences and perspectives associated with heritage tourism and indigenous well-being.

Intergenerational representation was deemed essential, as much traditional knowledge transmission and emotional support systems occur through elder-younger generation interactions (Yang and Warburton, 2018). Older participants shared their insights on traditions, healing and cultural continuity, whereas younger participants discussed identity formation, tourism employment, cultural adaptation and psychosocial stressors associated with modernization. There was an effort to maintain gender balance within the sample, as women engaged in handicraft production, storytelling traditions and community rituals provided important perspectives regarding emotional support systems, group identity and economic benefits associated with tourism participation. Participation was voluntary and participants were informed of the research purposes, confidentiality procedures and their right to withdraw from the study at any stage. This resulted in a culturally authentic and diverse sample representing a broad range of psychosocial experiences relevant to the study objectives (Yang and Warburton, 2018; Kohsaka and Rogel, 2021).

### 2.4 Data collection and psychosocial assessment

Qualitative data were collected through semi-structured interviews with elders, traditional healers, tourism organizers, artisans and cultural practitioners to explore themes related to culture-based identity, emotional healing, ritual participation, ecological spirituality, opportunities associated with heritage tourism and psychosocial challenges experienced within indigenous communities (Semenya and Potgieter, 2014). Quantitative data were obtained using structured psychosocial questionnaires designed to assess emotional well-being, resilience, stress perception and social connectedness (Brooks et al., 2023). A significant component of the ethnographic methodology involved participant observation, including attendance at cultural festivals, healing rituals, storytelling gatherings, dance performances, eco-cultural tours and communal events to document emotional expressions, interpersonal interactions and participation in collective cultural activities. Observational field notes captured symbolic meanings, ritual practices, social relationships and visitor–community dynamics associated with heritage tourism. Table 2 shows reliability statistics of psychosocial measures.

**Table 2:** Reliability statistics of psychosocial measures.

Instrument	Items	Cronbach’s α
CD-RISC-10	10	0.89
WHO-5	5	0.91
MSPSS	12	0.88
PSS-10	10	0.86
Cultural identity scale	8	0.90

Focus group discussions were conducted with elders, women and youth participants to examine perceptions of emotional well-being, cultural continuity, tourism participation and social transformation, including themes of identity pride, communal solidarity, emotional resilience and cultural commodification (Kang and Song, 2021). Structured psychosocial questionnaires included culturally adapted measures of emotional stability, perceived social support, resilience, cultural belongingness and stress reduction associated with participation in heritage tourism activities. Psychological well-being and psychosocial outcomes were assessed using culturally adapted and validated instruments, with cultural adaptation undertaken through consultation with community representatives and bilingual cultural mediators to ensure conceptual relevance and linguistic appropriateness. The assessment framework incorporated the Connor–Davidson Resilience Scale (CD-RISC-10) to evaluate psychological resilience, the WHO-5 Well-Being Index to measure emotional well-being, the Multidimensional Scale of Perceived Social Support (MSPSS) to assess social connectedness and perceived support and the Perceived Stress Scale (PSS-10) to evaluate stress perception. Cultural identity and community belongingness were assessed using an adapted version of the Cultural Identity and Belongingness Scale (CIBS), a multidimensional instrument developed to evaluate individuals’ sense of cultural affiliation, attachment to their community, and perceived cultural continuity. Because the original instrument was designed for culturally diverse populations, minor contextual adaptations were undertaken to ensure its suitability for indigenous heritage tourism settings. The adaptation process involved expert consultation with two psychologists, two indigenous cultural scholars, and one tourism specialist to ensure cultural relevance while maintaining the original conceptual domains. The final instrument consisted of 12 items rated on a five-point Likert scale ranging from 1 (strongly disagree) to 5 (strongly agree), with higher scores indicating stronger cultural identity and community belongingness. Content validity was established through expert review, yielding a Content Validity Index (CVI) of 0.91. Internal consistency demonstrated excellent reliability (Cronbach’s *α* = 0.89), while composite reliability was 0.91. Prior to the main survey, the adapted questionnaire was pilot-tested among 30 participants from a neighboring indigenous community to confirm clarity, cultural appropriateness, and linguistic consistency (Brooks et al., 2023; Kang and Song, 2021).

### 2.5 Ethical considerations and cultural sensitivity

Ethical approval for the study was obtained from the Institutional Research Ethics Committee of Wenshan University and the approval number is “WSXY2024112.” Community representatives were actively involved throughout the research process, including study design, interpretation of findings and dissemination of results, ensuring adherence to principles of Indigenous self-determination and collaborative research governance. Ethical sensitivity and cultural respect were prioritized throughout the research process, with ethical approval for the study granted through the appropriate institutional review framework and further permissions granted through community leadership councils and cultural representatives before data collection (Celidwen et al., 2023). Traditional cultural practices often hold sacred knowledge, ritual limitations and communal stewardship; therefore, researchers respected elders and cultural custodians and avoided recording certain ceremonies identified by communities as being spiritually restricted or otherwise culturally sensitive.

Participants were given both verbal and written explanations of the purpose of the study, data collection procedures, confidentiality measures and expected publication outcomes (Colom and Rohloff, 2018). All participation was voluntary and participants had the right to withdraw from the study at any time without penalty. The data were transcribed using coded identifiers and analyses were conducted using pseudonyms rather than participant names and, where necessary, without identifying specific communities to further protect confidentiality.

Communities participating in the study were encouraged to contribute contextual insights throughout the research process and were provided opportunities to review preliminary interpretations where appropriate. Feedback from community representatives assisted in clarifying issues related to cultural loss, identity conflict and social marginalization associated with tourism development. Sensitive topics were approached with caution to avoid psychological distress and to protect the collective well-being of participants. Individuals were free to decline any questions they considered culturally sensitive or personally uncomfortable. This collaborative and participatory approach ensured that ethical practice extended beyond procedural approval to encompass respect for Indigenous dignity, cultural autonomy, collective rights and community ownership of cultural knowledge, consistent with culturally responsive and community-engaged research principles (Celidwen et al., 2023; Colom and Rohloff, 2018).

### 2.6 Data analysis and interpretation

An integrated thematic and descriptive analytical approach was employed to analyze the psychosocial impact of heritage tourism on indigenous communities using both qualitative and quantitative data collected during the study (Shabalala, 2024). Qualitative materials were obtained from interviews, focus group discussions and observational field notes that were transcribed and systematically coded to identify recurring emotional, cultural and social themes (Ahmed et al., 2025). Qualitative data analysis followed a structured thematic analysis procedure involving familiarization with the data, initial code generation, theme development, theme review, theme definition and naming and final report production. Initial coding included expressions related to emotional healing, resilience, identity reinforcement, social cohesion, ecological attachment, ritual participation and tourism-related stress. Similar codes were subsequently organized into broader psychosocial themes reflecting collective experiences of mental wellness and cultural continuity.

Multiple researchers reviewed coded materials for analytical reliability and interpretive discrepancies were resolved through discussion and contextual verification, with reflexive notes incorporated into the analytical process to minimize researcher bias and maintain cultural authenticity (Yousefi Afrashteh, 2025). Interview transcripts were independently coded by two trained qualitative researchers and intercoder reliability assessment demonstrated an overall agreement rate of 91.4%. To further enhance credibility and cultural validity, member-checking procedures were conducted with community representatives who reviewed preliminary interpretations and provided feedback regarding cultural accuracy and contextual relevance. The final thematic framework identified six major themes: cultural participation and resilience, traditional healing and psychological wellness, social cohesion and belongingness, ecological attachment and spiritual restoration, economic empowerment and well-being and tourism-related stress and cultural commodification.

Quantitative data were analyzed using IBM SPSS Statistics Version 29.0 (IBM Corp., Armonk, NY, USA). Descriptive statistics were computed and presented as frequencies, percentages, means and standard deviations. Group differences in psychosocial variables across levels of heritage tourism participation were examined using one-way analysis of variance (ANOVA), followed by Tukey’s *post hoc* comparisons where the significant differences were identified. Pearson’s correlation analysis was conducted to examine relationships among cultural participation, emotional resilience, psychological stability, cultural identity and community belongingness. Multiple linear regression analysis was subsequently performed to determine the independent contribution of heritage tourism participation to psychological well-being after controlling for relevant demographic variables. Prior to regression analysis, assumptions of normality, linearity, homoscedasticity, independence of residuals and multicollinearity were evaluated. Residual plots confirmed acceptable homoscedasticity and linearity, the Durbin–Watson statistic indicated independence of residuals and variance inflation factor (VIF) values below 5 confirmed the absence of multicollinearity. Statistical significance was established at *p* < 0.05 (Shabalala, 2024; Ahmed et al., 2025; Yousefi Afrashteh, 2025).

## 3 Results

### 3.1 Cultural participation and emotional resilience

Participants reported that participation in heritage tourism activities, including traditional ceremonies, storytelling circles, music and dance events and other shared rituals were emotionally rejuvenating and helped to maintain a sense of inner harmony and psychological resilience. Quantitative analysis further demonstrated that heritage tourism participation was positively associated with psychological well-being, resilience and cultural identity. Correlation analysis revealed a significant positive relationship between heritage tourism participation and psychological well-being (*r* = 0.38, *p* < 0.01), resilience (*r* = 0.31, *p* < 0.01), social support (*r* = 0.28, *p* < 0.01) and cultural identity (*r* = 0.34, *p* < 0.01). Participants with higher levels of cultural participation consistently reported stronger emotional resilience and lower perceptions of social isolation than those with limited participation in cultural tourism activities.

Community elders emphasized that cultural participation was a bridge between generations and reduced social isolation and identity loss and cultural practices associated with seasonal festivals, spiritual cleansing and ecological worship were referenced as a source of emotional healing and stress reduction and group singing, drumming and ceremonial dance were described as releasing emotion and reaffirming group cohesion in times of suffering. Interview findings supported these quantitative observations. One Miao elder explained that participation in community festivals strengthened emotional well-being by reinforcing ancestral connections and collective identity. Similarly, a young Dong participant reported that traditional dance performances enhanced self-confidence, reduced emotional stress and strengthened cultural pride. These narratives suggest that cultural participation functions not only as a heritage preservation activity but also as an important psychosocial resource. Table 3 presents psychosocial indicators associated with cultural participation in heritage tourism.

**Table 3:** Psychosocial indicators associated with cultural participation in heritage tourism.

Cultural activity	Major psychological benefit	Community response
Storytelling sessions	Emotional expression and identity reinforcement	Increased intergenerational bonding
Traditional dance	Stress reduction and social engagement	Enhanced cultural pride
Ritual ceremonies	Emotional resilience and spiritual comfort	Strong collective participation
Music and drumming	Emotional release and relaxation	Improved communal solidarity
Craft-making workshops	Confidence and social belongingness	Greater women’s participation

Thematic analysis identified two dominant subthemes within this category: (1) identity reinforcement and (2) emotional coping. Participants frequently linked cultural participation with a stronger sense of belonging, cultural continuity and collective resilience. The findings therefore indicate convergence between quantitative evidence and qualitative narratives, highlighting the role of heritage tourism in promoting emotional regulation, psychological resilience and community well-being.

*“When we gather for our ceremonies, we remember who we are. Our traditions give us strength during the difficult times and help us face life’s challenges together.”* (Participant 18, Female, 47 years).

**Figure 1:**
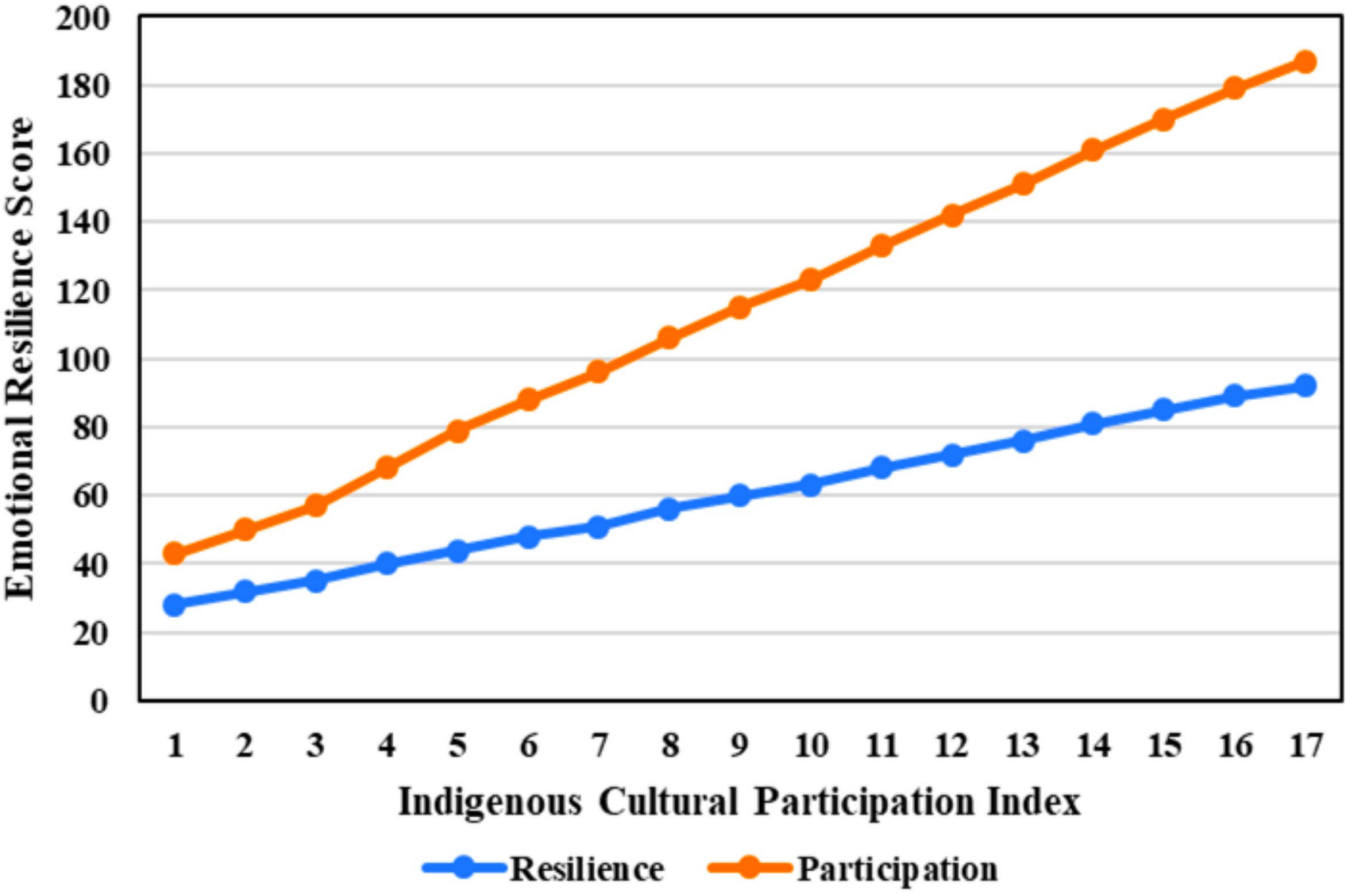
Relationship between indigenous cultural participation and emotional resilience.

Participants with a cultural tourism involvement were found to have higher resilience scores than participants who were minimally involved with tourism-related cultural engagement, who attributed these benefits to social interaction, recognition of indigenous identity and preservation of ancestral knowledge and who noted that performing traditional arts for visitors made them feel more confident and less marginalized culturally. Respondents to heritage tourism survey questions reported that restoring lost traditions and hosting consistent community activities enhanced access to social support and social connectedness and women in cultural groups reported that cooperative crafting and narrative circles provided venues for sharing personal experiences and managing stress, even if some felt emotionally drained from performing and having to meet outside expectations during busy tourism times. Figure 1 shows relationship between indigenous cultural participation and emotional resilience.

### 3.2 Traditional healing practices and psychological wellness

Traditional healing practices emerged as an important contributor to psychological wellness within the participating indigenous communities. Participants described healing systems incorporating spirituality, herbal medicine, ritual purification, ancestral guidance, ecological interaction, music and prayer, which were frequently integrated into heritage tourism activities. Quantitative findings indicated that participants who regularly engaged in traditional healing practices reported lower perceived stress and higher psychological well-being scores than those with limited participation. Furthermore, cultural identity demonstrated a significant positive association with psychological well-being (*r* = 0.51, *p* < 0.01), suggesting that engagement in culturally embedded healing traditions contributed to emotional stability and resilience. Interview narratives supported these findings. Participants explained that healing rituals helped restore harmony between individuals, communities, nature and ancestral traditions. Traditional healers emphasized that emotional distress was often interpreted as an imbalance in social, spiritual and ecological relationships rather than solely an individual condition. Table 4 represents indigenous healing practices and associated mental wellness outcomes.

**Table 4:** Indigenous healing practices and associated mental wellness outcomes.

Traditional healing practice	Psychological outcome	Observed community impact
Herbal healing rituals	Emotional calmness	Greater trust in traditional medicine
Sacred smoke ceremonies	Stress reduction	Enhanced spiritual engagement
Forest meditation	Relaxation and emotional clarity	Increased ecological attachment
Ancestral chanting	Psychological comfort	Strengthened cultural continuity
River purification rituals	Emotional cleansing	Collective spiritual participation

Despite these benefits, participants expressed concerns regarding the commercialization of sacred healing practices for tourism purposes. Excessive visitor exposure was perceived as potentially reducing the spiritual significance of rituals and weakening community control over sacred knowledge.

*“The forest is our medicine. Walking among the sacred trees calms my mind more than any hospital because it reminds me of my ancestors.”* (Participant 36, Traditional Healer).

Thematic analysis identified two dominant subthemes spiritual healing and stress reduction indicating that indigenous healing systems functioned as culturally grounded psychosocial support mechanisms while simultaneously facing pressures associated with tourism commercialization. The qualitative narratives complemented these quantitative observations by illustrating how traditional healing practices extend beyond physical care to encompass emotional restoration, spiritual reassurance, and community connectedness. Participants frequently described ritual healing as a culturally meaningful process that reduced psychological distress while reinforcing relationships with ancestors, nature and community members.

### 3.3 Social cohesion and community belongingness

Heritage tourism was found to strengthen social cohesion and community belongingness by creating opportunities for collective cultural participation and intergenerational interaction. Participants described tourism-related activities, including cultural festivals, storytelling gatherings, handicraft exhibitions, traditional cooking events and youth volunteering programs, as important spaces for social engagement and mutual support. Quantitative findings revealed a significant positive association between social support and psychological well-being (*r* = 0.46, *p* < 0.01), while social support was also positively correlated with resilience (*r* = 0.52, *p* < 0.01). These findings indicate that stronger social networks contributed to enhanced emotional well-being and adaptive coping capacities among participants. Table 5 shows community-based tourism activities and social wellness indicators.

**Table 5:** Community-based tourism activities and social wellness indicators.

Tourism activity	Social wellness outcome	Community observation
Cultural festivals	Increased social cohesion	Strong communal participation
Storytelling circles	Intergenerational bonding	Enhanced cultural continuity
Handicraft cooperatives	Mutual emotional support	Greater women’s collaboration
Traditional cooking events	Collective identity strengthening	Improved social interaction
Youth tourism volunteering	Community responsibility	Reduced social isolation

**Figure 2:**
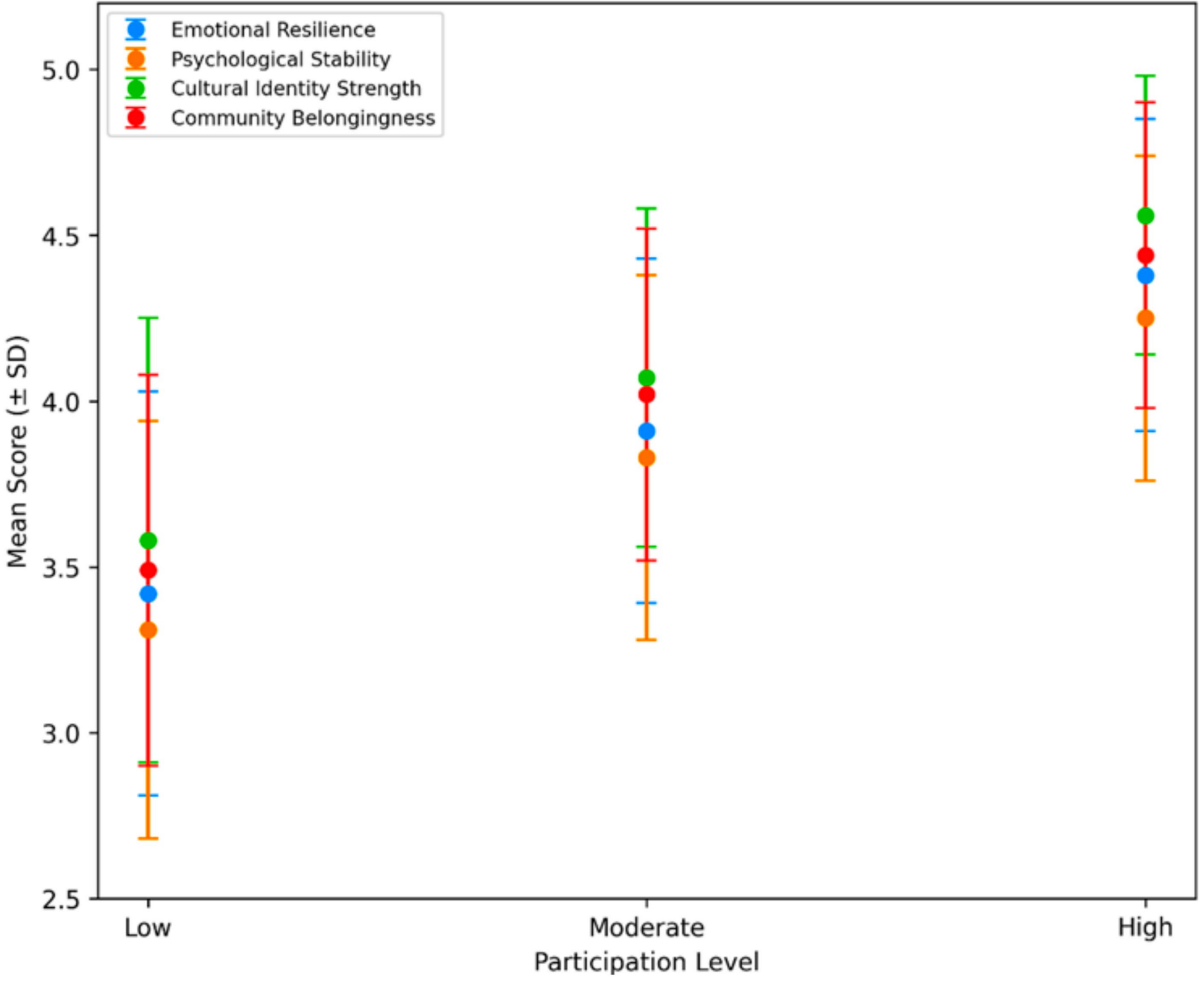
Psychosocial well-being indicators across heritage tourism participation levels.

Interview narratives further supported these findings. Community leaders reported that tourism planning and festival organization encouraged cooperation among households, while elders emphasized that storytelling traditions facilitated the transmission of cultural knowledge and emotional support across generations. Women participating in handicraft cooperatives highlighted the value of shared workspaces for discussing personal challenges and reducing stress. Figure 2 presents psychosocial well-being indicators across heritage tourism participation levels. Correlation matrix among study variables are given in Table 6.

**Table 6:** Correlation matrix among study variables.

Variable	1	2	3	4	5
Heritage tourism participation	1				
Cultural identity	0.34**	1			
Social support	0.28**	0.47**	1		
Resilience	0.31**	0.58**	0.52**	1	
Psychological well-being	0.38**	0.51**	0.46**	0.63**	1

*indicate statistical significance at *p* < 0.01 (two-tailed).

**Figure 3:**
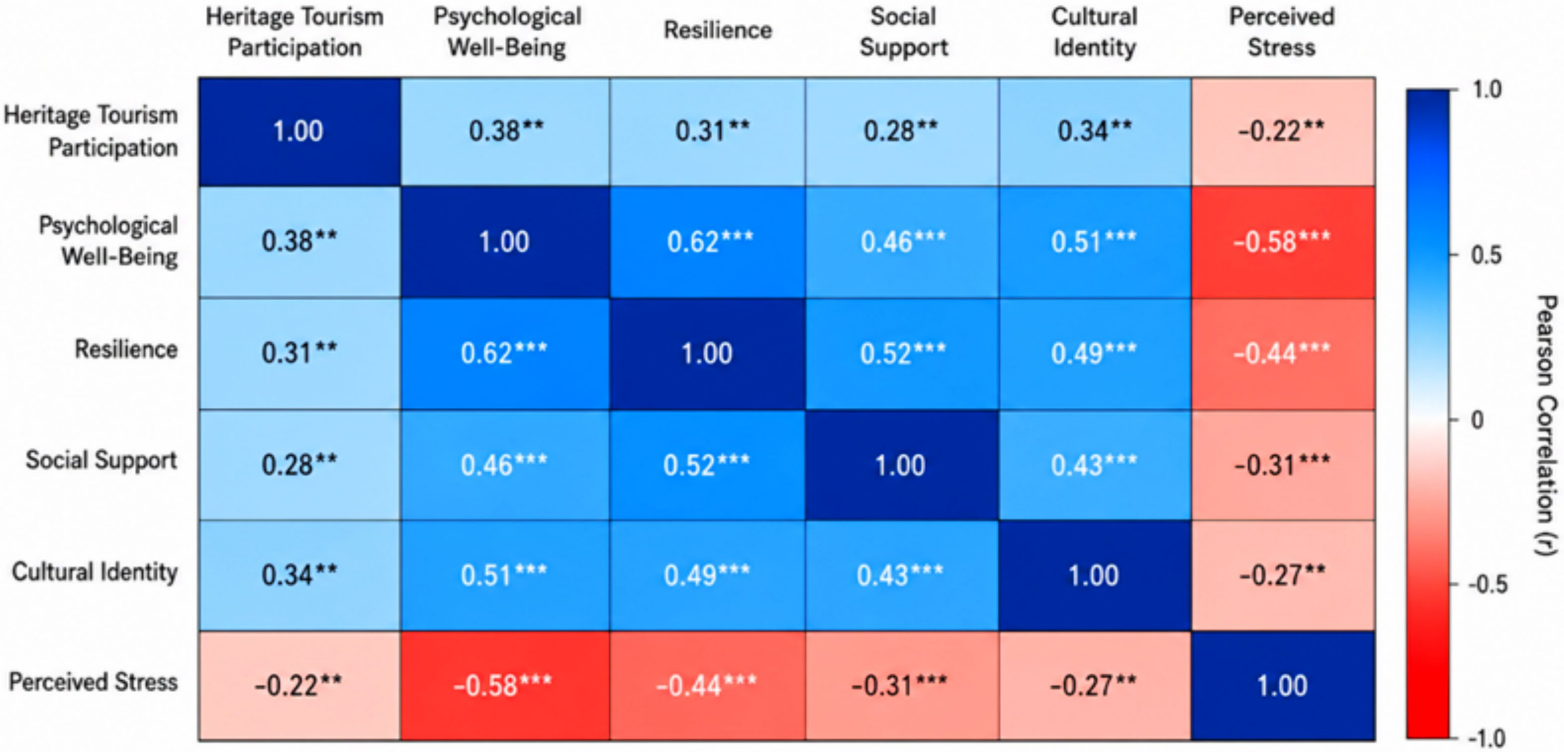
Correlation heatmap with relationships among key study variables.

Figure 3 presents correlation heatmap with relationships among key study variables. Youth participants also described increased cultural pride and stronger connections to family history through involvement in tourism-related activities. Thematic analysis identified social support and intergenerational bonding as the dominant subthemes within this category. Integration of quantitative and qualitative findings suggests that heritage tourism contributed positively to social connectedness, collective identity, interpersonal trust and community belongingness, although some concerns regarding income distribution and leadership roles were reported.

*“Tourism has brought our young people back to work with the elders. We now share stories and traditions together again.”* (Participant 52, Community Elder).

The qualitative findings further explained the quantitative improvements observed in social cohesion and community belongingness. Participants emphasized that collaborative tourism activities created opportunities for intergenerational learning, strengthened interpersonal relationships, and reinforced collective identity. These narratives provide contextual understanding of the higher community belongingness scores observed among participants with greater tourism involvement.

### 3.4 Ecological attachment and spiritual restoration

The findings revealed that ecological attachment and spiritual restoration were closely associated with heritage tourism participation and psychological well-being. Participants identified forests, rivers, mountains, agricultural lands and sacred ceremonial sites as culturally significant environments that fostered emotional peace, spiritual renewal and ancestral connection. Quantitative analysis demonstrated positive associations between cultural identity, resilience and psychological well-being, suggesting that engagement with culturally meaningful landscapes contributed to emotional stability and stress reduction. Participants who frequently engaged in eco-cultural activities reported greater feelings of relaxation, purpose and connection to their cultural heritage. Ecological tourism practices and psychological restoration indicators is presented in Table 7.

**Table 7:** Ecological tourism practices and psychological restoration indicators.

Ecological practice	Mental wellness benefit	Community observation
Sacred forest walks	Emotional relaxation	Enhanced spiritual awareness
River rituals	Stress reduction	Collective spiritual participation
Traditional farming	Psychological stability	Increased environmental responsibility
Medicinal plant education	Emotional confidence	Preservation of indigenous knowledge
Sacred landscape conservation	Emotional attachment	Greater ecological stewardship

Interview narratives highlighted the importance of ancestral lands in maintaining emotional well-being. Participants described sacred forests and rivers as places of reflection, healing and spiritual balance, while elders emphasized that traditional ecological knowledge encouraged respect, reciprocity and harmony with nature. Community members involved in eco-cultural tourism also reported a sense of pride when sharing indigenous environmental knowledge with visitors, reinforcing cultural identity and environmental stewardship. Thematic analysis identified sacred landscape attachment and environmental stewardship as the dominant subthemes within this category. The integration of quantitative and qualitative findings suggests that ecological participation and cultural engagement with natural environments contributed positively to psychological restoration, emotional resilience and spiritual well-being, while also encouraging community-led conservation efforts.

*“When I sit beside the sacred river, I feel peace. Visitors see only water, but for us it is a place where the mind becomes quiet.”* (Participant 71, Male, 55 years).

Qualitative interviews consistently highlighted the therapeutic importance of sacred landscapes for emotional restoration. Participants viewed forests, rivers and ceremonial grounds not only as tourism destinations but also as spiritually significant places where emotional healing and psychological balance were achieved. These observations support the quantitative findings demonstrating positive associations between ecological attachment and mental wellness.

### 3.5 Economic empowerment and mental well-being

The findings indicated that heritage tourism generated important economic opportunities that contributed to psychological well-being among indigenous community members. Participants reported that income derived from handicraft production, cultural performances, tourism guiding, traditional food enterprises and eco-cultural activities reduced financial insecurity and enhanced confidence, autonomy and future aspirations. Quantitative findings further indicated that heritage tourism participation was positively associated with psychological well-being (*r* = 0.38, *p* < 0.01) and cultural identity (*r* = 0.34, *p* < 0.01), suggesting that economic benefits were closely linked with improved psychosocial outcomes and strengthened cultural pride. Table 8 illustrates economic benefits of heritage tourism and associated mental health outcomes.

**Table 8:** Economic benefits of heritage tourism and associated mental health outcomes.

Economic activity	Psychological benefit	Social outcome
Handicraft sales	Improved self-esteem	Women’s empowerment
Cultural guiding	Identity affirmation	Youth engagement
Eco-tourism employment	Reduced economic stress	Community participation
Traditional food enterprises	Emotional confidence	Household stability
Cultural festival income	Social optimism	Community development

**Figure 4:**
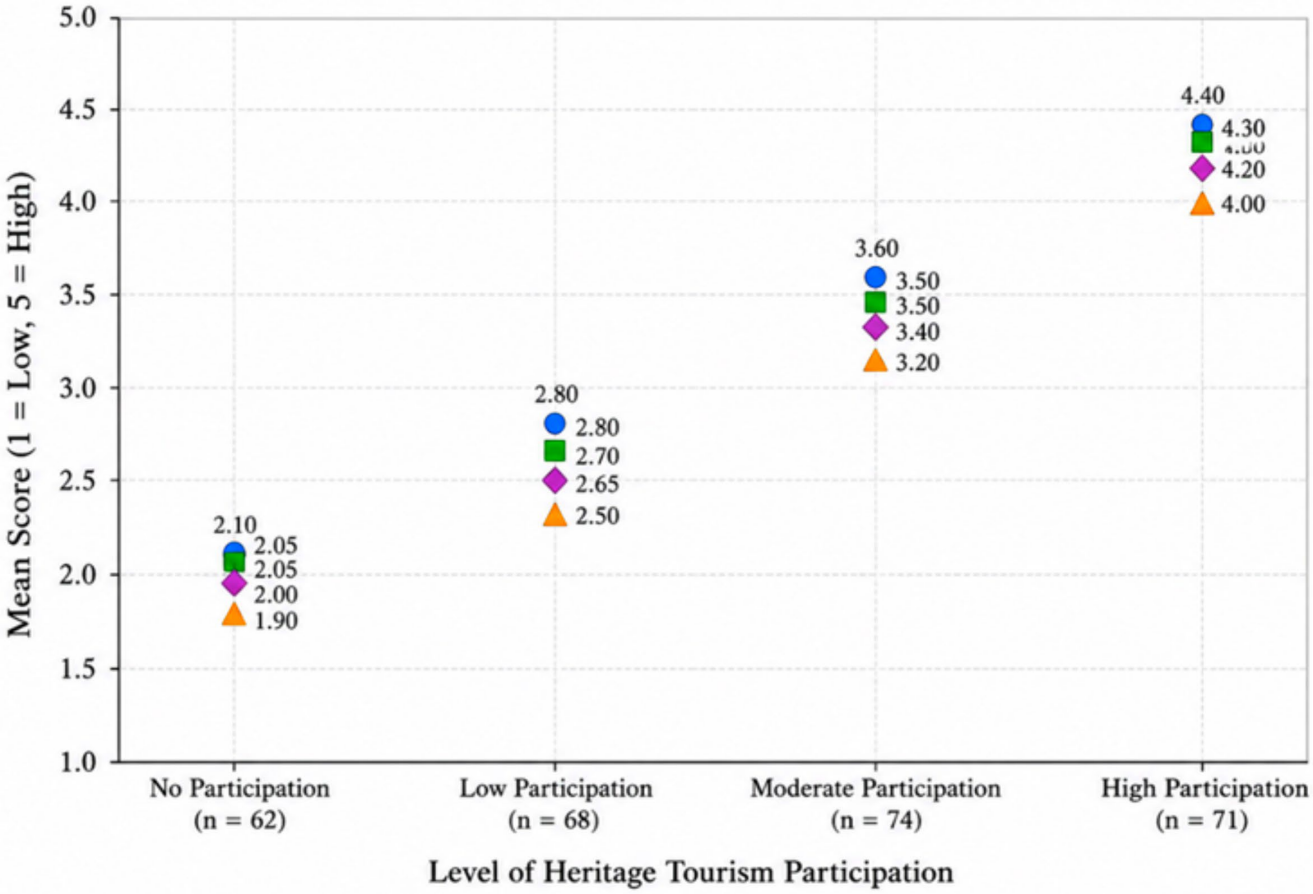
Psychosocial outcomes across levels of heritage tourism participation.

Interview narratives revealed that women involved in handicraft cooperatives experienced greater self-confidence and social recognition through their financial contributions to household livelihoods. Similarly, youth participants described tourism employment as a source of motivation and cultural pride, while community leaders emphasized that tourism-generated revenues supported local education, cultural festivals, environmental conservation and community development initiatives. Participants also reported that improved household income reduced family stress and enhanced social participation. Figure 4 illustrates psychosocial outcomes across levels of heritage tourism participation.

*“Selling our traditional handicrafts allows me to support my family while preserving our culture. It gives me confidence and hope for my children’s future.”* (Participant 88, Female Artisan).

Thematic analysis identified income generation and personal empowerment as the dominant subthemes within this category. Although concerns regarding seasonal income fluctuations and unequal benefit distribution were occasionally reported, the integration of quantitative and qualitative findings suggests that heritage tourism contributed positively to emotional well-being, self-efficacy and community optimism when managed through culturally sensitive and community-oriented development approaches. Interview narratives further demonstrated that the psychosocial benefits associated with heritage tourism extended beyond financial improvement. Participants frequently explained that economic opportunities increased self-confidence, family stability, and optimism while allowing them to preserve traditional cultural practices. These qualitative perspectives provide meaningful explanation for the higher psychological well-being scores observed among individuals with greater tourism participation.

### 3.6 Tourism-related stress and cultural commodification

Although heritage tourism was associated with numerous psychosocial benefits, participants also identified challenges related to tourism-related stress and cultural commodification. Community members reported concerns regarding increasing visitor expectations, commercialization of sacred traditions, performance pressures and unequal distribution of tourism benefits. Quantitative findings indicated that perceived stress remained negatively associated with psychological well-being, suggesting that tourism-related pressures may partially offset some of the positive psychosocial outcomes associated with cultural participation. Participants with intensive involvement in tourism activities reported higher levels of emotional fatigue during peak tourism seasons, particularly when cultural performances were required repeatedly for visitors. Table 9 presents negative psychosocial impacts associated with heritage tourism commercialization.

**Table 9:** Negative psychosocial impacts associated with heritage tourism commercialization.

Tourism-related challenge	Psychological impact	Community concern
Ritual commercialization	Emotional dissatisfaction	Loss of spiritual authenticity
Tourist overcrowding	Stress and fatigue	Environmental disturbance
Cultural performance pressure	Emotional exhaustion	Identity conflict
Unequal income distribution	Social tension	Community fragmentation
External tourism control	Psychological frustration	Reduced indigenous autonomy

Interview narratives revealed concerns that some sacred ceremonies were increasingly modified to satisfy tourist expectations, potentially weakening their spiritual significance and cultural authenticity. Participants also reported frustration when visitors displayed disrespectful behavior in sacred spaces, including inappropriate photography, environmental disturbance and disregard for local customs. Younger participants occasionally described tensions between maintaining authentic cultural identities and performing simplified cultural representations for tourism audiences. Table 10 represents multiple regression predicting psychological well-being.

**Table 10:** Multiple regression predicting psychological well-being.

Predictor	β	SE	t	*p*
Heritage tourism participation	0.19	0.05	3.82	<0.001
Cultural identity	0.29	0.06	4.76	<0.001
Social support	0.24	0.05	4.21	<0.001
Resilience	0.37	0.04	6.18	<0.001

*“Sometimes visitors only want photographs and performances. They do not understand that these ceremonies are sacred and have deep meaning for our people.”* (Participant 109, Male Ritual Practitioner).

Thematic analysis identified cultural commodification and tourism-related psychological pressure as the dominant subthemes within this category. Despite these concerns, many participants emphasized that community-led governance, visitor education programs, ethical tourism guidelines and culturally sensitive tourism policies could help mitigate negative impacts. Integration of quantitative and qualitative findings suggests that while heritage tourism can strengthen cultural pride and psychological well-being, sustainable benefits depend upon maintaining cultural integrity, equitable participation and indigenous control over tourism development processes.

## 4 Discussion

Research suggests that heritage tourism can positively contribute to mental health when practiced as a multidimensional cultural and psychosocial phenomenon that affords access to cultural continuity and the public affirmation of one’s own indigenous traditions (Allen et al., 2021), which can foster emotional resilience through social cohesion and identity reinforcement, as culturally grounded theories posit that emotional well-being is connected to group membership and belongingness rather than individual psychological functioning (Allen et al., 2021). The present findings support this perspective, as quantitative analyses demonstrated significant positive associations between heritage tourism participation, cultural identity, resilience, social support and psychological well-being. Qualitative narratives further revealed that participation in traditional ceremonies, storytelling activities, music, dance and ritual practices strengthened emotional coping, identity reinforcement and collective resilience among Miao, Dong and Naxi participants.

The significant strength of the present convergent mixed-methods design lies in the integration of quantitative psychosocial findings with qualitative participant narratives. While quantitative analyses demonstrated significant positive associations between heritage tourism participation and indicators of emotional resilience, psychological stability, cultural identity and community belongingness, the qualitative findings provided valuable contextual explanations for these relationships. Participants consistently described indigenous ceremonies, traditional healing practices, storytelling, and communal cultural activities as emotionally restorative experiences that fostered identity affirmation, collective support and psychological resilience. The convergence of both datasets strengthens confidence in the interpretation that heritage tourism contributes to mental wellness through interconnected cultural, social, and spiritual mechanisms rather than solely through economic benefits.

Another significant issue identified in this study was the role of traditional healing systems as culturally embedded psychosocial support mechanisms. Participants described spiritual, ecological, ritual and ancestral practices as important sources of emotional balance, stress reduction and psychological restoration. Young people who re-engaged with traditional knowledge through tourism-related cultural activities reported increased cultural pride and stronger intergenerational connections. The quantitative findings indicating lower perceived stress and higher well-being among participants actively engaged in traditional healing practices further reinforce the importance of indigenous knowledge systems in supporting mental wellness. Healers emphasized that culturally embedded healing systems should be understood not only as expressions of mental wellness but as integral components of culture itself (Bihari, 2023).

The third major psychosocial outcome associated with heritage tourism was enhanced social cohesion, characterized by increased interpersonal cooperation, intergenerational interaction, collective responsibility and social solidarity. Community-based tourism activities strengthened communal identity and contributed to public recognition of indigenous cultures, thereby counteracting historical marginalization and cultural invisibility (Taylor, 2017). Consistent with the quantitative results, social support emerged as a significant correlate of resilience and psychological well-being, while qualitative evidence highlighted the role of storytelling circles, cultural festivals, handicraft cooperatives and youth volunteering programs in strengthening belongingness and emotional security. These findings suggest that heritage tourism may function as a mechanism for community empowerment when grounded in participatory cultural practices.

The findings also underscore the role of ecological connection in indigenous mental health, with sacred landscapes, forests, rivers and ceremonial spaces associated with emotional healing and spiritual renewal. Tourism activities involving ecological education, sacred site visits, traditional farming practices and land-based cultural learning enhanced environmental stewardship and emotional attachment to ancestral territories. Participants consistently described sacred landscapes as sources of psychological restoration, while quantitative findings suggested that stronger cultural identity and ecological engagement were associated with greater emotional well-being and resilience. These findings align with eco-psychological frameworks emphasizing the relationship between environmental identity and mental health. Indigenous perspectives generally conceptualize human well-being as inseparable from ecological balance, highlighting the importance of protecting cultural landscapes within tourism development policies.

The positive psychological outcomes associated with heritage tourism were also linked to economic empowerment, including increased income-generating opportunities, enhanced financial independence among women and youth, reduced financial stress and improved self-esteem among those engaged in cultural enterprises (Aquino et al., 2018). Interview narratives demonstrated that tourism-related income increased confidence, autonomy and future optimism, while community-level benefits supported education, environmental conservation and cultural revitalization initiatives. However, the findings indicate that economic benefits alone are insufficient to guarantee positive mental health outcomes. Equitable distribution of tourism revenues, community participation in decision-making and respect for indigenous cultural autonomy remain essential to preventing social tension and psychological dissatisfaction.

The tension between cultural preservation and commercialization emerged as one of the most important findings of the study. Although tourism can contribute to the revitalization of cultural traditions and increased public awareness of indigenous heritage, excessive commodification may diminish the spiritual significance of cultural practices and generate emotional detachment as sacred traditions are transformed into tourism products (Kozinets and Borghini, 2013). The tourism-related stress theme identified in the present study revealed concerns regarding cultural performance pressure, emotional exhaustion, ritual commercialization and loss of authenticity. Participants who were highly engaged in tourism activities reported greater emotional fatigue during peak visitation periods and expressed concern about external influences on cultural representation. These findings suggest that culturally ethical and psychologically sustainable tourism frameworks are necessary to ensure that indigenous communities maintain control over cultural representation, sacred knowledge and tourism development processes.

This study contributes to the growing interdisciplinary literature spanning psychology, anthropology, tourism studies and indigenous knowledge systems by demonstrating that heritage tourism can function as a culturally grounded resource associated with resilience, belongingness, emotional healing and psychological well-being. A major strength of the present research lies in the convergence between quantitative and qualitative findings, which consistently indicated positive relationships among heritage tourism participation, cultural identity, social support, resilience and well-being while simultaneously identifying tourism-related stressors that require careful management. This mixed-methods evidence provides a more comprehensive understanding of how heritage tourism influences indigenous mental health than either qualitative or quantitative approaches alone.

The study nevertheless has several limitations. Existing indigenous communities already engaged in heritage tourism were examined and therefore the findings may not be transferable to all indigenous populations. Cultural experiences are context-dependent and may vary across geographical settings and historical periods. Furthermore, psychosocial outcomes were assessed through self-report measures and may be influenced by social desirability bias or culturally specific interpretations of survey items. The cross-sectional nature of the study also limits causal inference; therefore, the observed associations between heritage tourism participation and psychological well-being should not be interpreted as evidence of direct causation.

Future research should examine the long-term psychosocial impacts of heritage tourism across diverse cultural contexts and explore gender differences, youth identity formation and indigenous governance structures in greater depth. Longitudinal and comparative studies are particularly needed to determine how changing tourism dynamics influence resilience, cultural identity, and mental health outcomes over time. Greater integration of indigenous research methodologies and collaborative community partnerships would further strengthen future investigations into the mental health implications of heritage tourism.

## 5 Conclusion

The research investigated the potential impact of heritage tourism on mental wellness among indigenous communities within their traditional settings and concluded that heritage tourism may serve as a psychosocial resource contributing to emotional resilience, collective identity, social cohesion and spiritual well-being while indigenous cultural practices including storytelling, rituals, music, dance, ecological stewardship and healing traditions contribute significantly toward psychological stability and emotional restoration.

Heritage tourism also increased cultural pride and sense of belongingness among community members and promoted the intergenerational transmission of traditional knowledge; sacred landscapes and ecological relationships were found to be important factors in indigenous mental wellness, which showed the cultural significance of environmental conservation in cultural tourism; and economic opportunities created by tourism increased self-esteem, social participation and emotional security, especially for women and youth.

The convergent mixed-methods approach strengthened the credibility of the findings by integrating the quantitative psychosocial evidence with qualitative participant experiences. The consistency observed between the statistical outcomes and personal narratives demonstrates that indigenous cultural participation, traditional healing practices, ecological attachment and community engagement collectively promote emotional resilience, cultural identity and psychological well-being. These findings highlighted the importance of culturally grounded heritage tourism initiatives that preserve indigenous knowledge while simultaneously supporting sustainable mental health promotion and the community resilience.

## Data Availability

The original contributions presented in the study are included in the article/supplementary material, further inquiries can be directed to the corresponding author.
